# New Methods of Treating Hydrocele

**Published:** 1894-11

**Authors:** 


NEW METHODS OF TREATING HYDROCELE.—The classical method of treating hydrocele by injection of tincture of iodine, while often successful, is very painful, and one of the following three methods described in the *Dublin Journal* can often be employed with quite as good results:

(*a*) The first is that practiced by M. Neumann, who operates thus: He taps the sac with the usual trocar, and while the liquid is flowing out, he pushes in still deeper the canula and fixes it by a bandage in the parts, where he leaves it two days. In six cases thus treated the union of the internal walls was effected without inflammation. When the canula is withdrawn, compresses of lead lotion are applied, and the patient cured in about a week.

(*b*) The second is that adopted by Dr. Buschke. After disinfecting the part, he inserts a large sized trocar, and when the contents are removed, he injects a weak solution of phenic acid, which he allows to remain a few minutes, manipulating the parts in the meantime. The trocar is inserted a second time and the scrotum entirely transfixed from below upwards, the second opening being made in the superior portion of the sac. Through the canula is passed a fenestrated drainage-tube, and the instrument finally withdrawn; an aseptic bandage completes the operation. The patient can go about on the same day, and on the fourth or sixth day the drain is removed and a new dressing applied. Thirteen patients were treated in this way, with very satisfactory results.

(*c*) The third method is applied chiefly to children. A large curved needle, armed with silk thread, is passed through the sac from above downwards. A fine trocar draws off the liquid, and then the ends of the thread are tied above the scrotum, and the apertures carefully sealed up with collodion. The threads are removed at the end of six or eight days, and the apertures again stopped with collodion. By this time the patient may be considered as cured.—*Virginia Medical Monthly.*

